# Design and implementation of the Our Health Counts (OHC) methodology for First Nations, Inuit, and Metis (FNIM) health assessment and response in urban and related homelands

**DOI:** 10.17269/s41997-024-00867-9

**Published:** 2024-04-15

**Authors:** Janet Smylie, Cheryllee Bourgeois, Marcie Snyder, Raglan Maddox, Stephanie McConkey, Michael Rotondi, Conrad Prince, Brian Dokis, Michael Hardy, Serena Joseph, Amanda Kilabuk, Jo-Ann Mattina, Monica Cyr, Genevieve Blais

**Affiliations:** 1 Well Living House, Li Ka Shing Research Institute, Unity Health Toronto, Toronto, ON Canada; 2 Dalla Lana School of Public Health and Department of Family & Community Medicine, Faculty of Medicine, University of Toronto, Toronto, ON Canada; 3 Seventh Generation Midwives Toronto, Toronto, ON Canada; 4 National Centre for Epidemiology and Public Health, College of Health & Medicine, The Australian National University, Canberra, Australia; 5 School of Kinesiology and Health Science, York University, Toronto, ON Canada; 6 Women’s College Hospital, Toronto, ON Canada; 7 Southwest Ontario Aboriginal Health Access Centre, London, ON Canada; 8 Anishnawbe Mushkiki Aboriginal Health Access Centre, Thunder Bay, ON Canada; 9 Waasegiizhig Nanaandawe’iyewigamig Aboriginal Health Access Centre, Kenora, ON Canada; 10 Tungasuvvingat Inuit, Ottawa, ON Canada; 11 De dwa da dehs nye>s Aboriginal Health Centre, Hamilton, ON Canada; 12 Aboriginal Health & Wellness Centre, Winnipeg, MB Canada

**Keywords:** Indigenous research methodologies, Indigenous health assessment, Respondent-driven sampling, Indigenous health information systems, Urban Indigenous, Indigenous health survey, Indigenous data linkage, Méthodes de recherche autochtones, évaluation de la santé autochtone, échantillonnage en fonction des répondants, systèmes autochtones d’information sur la santé, Autochtones en milieu urbain, enquête de santé autochtone, couplage de données autochtones

## Abstract

**Objectives:**

Methods for enumeration and population-based health assessment for First Nations, Inuit, and Metis (FNIM) living in Canadian cities are underdeveloped, with resultant gaps in essential demographic, health, and health service access information. Our Health Counts (OHC) was designed to engage FNIM peoples in urban centres in “by community, for community” population health assessment and response.

**Methods:**

The OHC methodology was designed to advance Indigenous self-determination and FNIM data sovereignty in urban contexts through deliberate application of Indigenous principles and linked implementation strategies. Three interwoven principles (*good relationships are foundational*; *research as gift exchange*; and *research as a vehicle for Indigenous community resurgence*) provide the framework for linked implementation strategies which include actively building and maintaining relationships; meaningful Indigenous community guidance, leadership, and participation in all aspects of the project; transparent and equitable sharing of project resources and benefits; and technical innovations, including respondent-driven sampling, customized comprehensive health assessment surveys, and linkage to ICES data holdings to generate measures of health service use.

**Results:**

OHC has succeeded across six urban areas in Ontario to advance Indigenous data sovereignty and health assessment capacity; recruit and engage large population-representative cohorts of FNIM living in urban and related homelands; customize comprehensive health surveys and data linkages; generate previously unavailable population-based FNIM demographic, health, and social information; and translate results into enhanced policy, programming, and practice.

**Conclusion:**

The OHC methodology has been demonstrated as effective, culturally relevant, and scalable across diverse Ontario cities.

## Résumé

**Objectifs:**

Les méthodes de dénombrement et d’évaluation populationnelle de la santé des personnes des Premières Nations, des Inuits et des Métis (PNIM) vivant dans les villes du Canada sont sous-développées, ce qui laisse des lacunes dans les informations essentielles sur le profil démographique, la santé et l’accès aux services de santé. Le projet Notre santé compte (NSC) vise à collaborer avec les personnes des PNIM dans les centres urbains au moyen d’une évaluation de la santé des populations et d’une intervention « pour nous-mêmes, par nous-mêmes ».

**Méthode:**

La méthode NSC est conçue pour renforcer l’autodétermination autochtone et la souveraineté des données des PNIM vivant en milieu urbain par l’application délibérée de principes autochtones et de stratégies de mise en œuvre connexes. Trois principes imbriqués (« les bonnes relations sont fondamentales »; « la recherche en tant qu’échange de cadeaux »; et « la recherche comme vecteur de résurgence des communautés autochtones ») constituent le cadre de stratégies de mise en œuvre connexes : l’établissement et le maintien actifs de relations; la guidance, la participation et le leadership significatifs des communautés autochtones dans tous les aspects du projet; le partage transparent et équitable des ressources et des avantages du projet; et les innovations techniques, dont l’échantillonnage en fonction des répondants, les enquêtes de santé exhaustives et personnalisées, et les couplages avec les fonds de données de l’Institut de recherche en services de santé (ICES), pour produire des indicateurs d’utilisation des services de santé.

**Résultats:**

L’approche NSC a réussi dans six agglomérations urbaines de l’Ontario : à renforcer la souveraineté des données et la capacité d’évaluation de la santé des populations autochtones; à recruter et à mobiliser de vastes cohortes représentatives des PNIM vivant en milieu urbain et sur les territoires connexes; à personnaliser des enquêtes de santé exhaustives et des couplages de données; à générer des informations démographiques, sanitaires et sociales non disponibles auparavant sur les populations des PNIM; et à traduire ces résultats en politiques, en programmes et en pratiques améliorés.

**Conclusion:**

Il est démontré que la méthode NSC est efficace, culturellement appropriée et modulable dans différentes villes de l’Ontario.

## Introduction

Indigenous peoples have resided on the lands currently identified as Canadian^1^ cities for dozens of millennia. Without exception, these cities are located on traditional First Nations, Inuit, and/or Metis territories. Colonial policies of forced relocation of First Nations, Inuit, and Metis (FNIM) peoples and restricted access of “Status Indians” and Metis people to participate in urban development contributed to persistent narratives that these lands were designated for colonists and devoid of Indigenous peoples (Dorries, 2022). Despite these limiting policies, there are examples of Indigenous peoples playing important roles in the historic development of Canadian cities, for example the role and contributions of Metis people in the inception and historic development of Winnipeg (Peterson, 1985; Peters et al., 2018) or the construction of Canadian bridges and subsequently American skyscrapers by Mohawk ironworkers (Noble, 2023).

Recent decades have seen an accelerated growth of FNIM populations in urban homelands of Canada and Indigenous populations globally (Statistics Canada, 2022a; National Association of Friendship Centres, 2021; United Nations, 2007). This has been accompanied by a proliferation of Indigenous health and social service providers in urban settings, including dedicated urban Indigenous health access centres (Alliance for Healthier Communities, 2023; Aboriginal Health and Wellness Centre of Winnipeg, 2023). According to the 2021 Census, 60% of FNIM live in urban centres of at least 30,000 (Statistics Canada, 2022a, 2022b). In the province of Ontario, according to the Census, more than 70% of FNIM currently live in urban areas (Statistics Canada, 2022b). Evidence unmasking census undercounting of FNIM living in cities indicates that the actual numbers of FNIM living in urban areas are significantly higher than those reported by the Census (Smylie et al., 2018; Rotondi et al., 2017; Smylie & Firestone, 2015).

Recognizing and responding to the needs of the vibrant and diverse FNIM populations in urban homelands across Canada are powerfully impeded by historic and current colonial policies designed to segregate, control, and assimilate FNIM peoples (Dorries, 2022). Urban health and social services systems have been primarily designed to respond to the social and economic needs of non-Indigenous settlers living on appropriated homelands (Dorries, 2022; Collier, 2020; Health Council of Canada, 2012). Federal and provincial governmental engagement and policies are commonly focused on the engagement of and response to FN living in on-reserve communities; and Inuit in land claim areas (Collier, 2020). With some exceptions, FNIM living in urban and related homelands in Canada are commonly discounted in population enumeration (i.e. the process of identifying and counting all individuals in a given geography or social group), needs assessment, and linked policies, programming, and services agreements (Smylie & Firestone, 2015; Collier, 2020). In contrast, governmental policies and practices in Australia and New Zealand commonly include Indigenous peoples living in cities.

The tangible harms of this exclusionary approach not only to FNIM living in cities, but also to their relatives living in rural and remote homelands, have been highlighted during the COVID-19 pandemic. FNIM living in cities were commonly the first Indigenous populations geographically to be exposed to SARS-COV-2; experienced disproportionate rates of SARS-COV-2 infection and hospitalization compared to non-Indigenous urban and non-urban Indigenous populations; and faced barriers in access to testing and vaccination with subsequently lower rates of vaccine uptake (Smylie et al., 2022). Further, early outbreaks in First Nations on-reserve communities were commonly linked to exposure from a relative who had travelled from the city.

One important consequence of persistent colonial policies and approaches, such as those presented above, is that existing systems of enumeration and population-based health assessment for FNIM living in urban areas remain strikingly inadequate (Collier, 2020; Smylie & Firestone, 2015). Gaps in essential demographic, health, and health service access information perpetuate the discounting of FNIM living in urban and related homelands and mask the ongoing unequal distribution of health and social resources and linked health inequities.

The overarching goal of every Our Health Counts (OHC) study is to address these data gaps while engaging FNIM peoples who reside in urban centres in “by community, for community” population health assessment and response. To date, we have successfully implemented six OHC studies across Ontario, Canada: OHC First Nations Hamilton (Firestone et al., 2014; Smylie et al., 2011), OHC Inuit Ottawa (Smylie & Firestone, 2017; Smylie et al., 2018), OHC Toronto (Rotondi et al., 2017; Kitching et al., 2020; Well Living House, 2023a), OHC London (Smylie et al., 2022; Southwest Ontario Aboriginal Health Access Centre, 2023), OHC Thunder Bay (Well Living House, 2023b), and OHC Kenora (McConkey et al., 2022; Snyder et al., 2022a, 2022b, 2022c). A seventh is in progress in Manitoba, Canada (OHC Winnipeg). The purpose of this paper is to provide an in-depth description of the OHC methodology. This includes our Indigenous conceptualization of how and why this methodology works; key principles and linked implementation strategies; and cross-cutting Indigenous health information, community well-being, health service, and health policy impacts.

In order to avoid an essentialized pan-Indigenous discussion, we note that the following articulation of OHC methodology relies heavily on the specific Metis-Cree worldviews of the two first authors, who are themselves Metis-Cree. Our hope is that by applying these specific Metis-Cree concepts and language, we will stimulate additional nation-specific methodology discussions and dialogue that assert diverse FNIM and international Indigenous paradigms and languages.

## Methodology

The OHC research methodology has been iteratively developed over time by Indigenous health scholars working in partnership with urban Indigenous service providers (Table 1). Together, we have purposefully designed OHC studies to advance Indigenous self-determination and FNIM data sovereignty in urban contexts. Our methodology moves beyond, and is distinct from, community-based participatory action research in its deliberate application and demonstration of Indigenous paradigms, principles, knowledge, and practices. The Indigenous-led academic research team and Indigenous service provider partners share pre-existing commitments to and practical experience in advancing FNIM rights in cities and beyond, including the right to access culturally safe health and social services. The OHC Indigenous community‒partnered research approach has been shaped by local Indigenous activism and the broader domestic (Truth and Reconciliation Commission of Canada, 2015; National Inquiry into Missing and Murdered Indigenous Women and Girls, 2019) and international Indigenous rights and reconciliation movements (United Nations General Assembly, 2007). The First Nations Regional Health Survey, which is focused on the population health assessment of First Nations living on-reserve, provided an inspirational and practical model of how to embed Indigenous authority, leadership, participation, and understandings of well-being into the quantitative health assessment domain (First Nations Regional Longitudinal Health Survey, 2002; The First Nations Information Governance Centre, 2019).

**Table 1:** Our Health Counts Indigenous health and social service provider core partners

OHC project	Local Indigenous service provider core partners	Provincial organizations co-governing OHC Hamilton and Ottawa
Hamilton First Nations	De dwa da dehs nye>s Aboriginal Health Centre	• Ontario Federation of Indian Friendship Centre • Ontario Native Women’s Association • Metis Nation of Ontario
Ottawa Inuit	Tungasuvvingat Inuit	
Toronto	Seventh Generation Midwives Toronto	
London	Southwest Ontario Aboriginal Health Access Centre	
Thunder Bay	Anishnawbe Mushkiki – Aboriginal Health Access Centre	
Kenora	Waasegiizhig Nanaandawe’iyeigamamig – Aboriginal Health Access Centre	

### OHC Conceptual Framework: community investment-ownership-activation

**Fig. 1:**
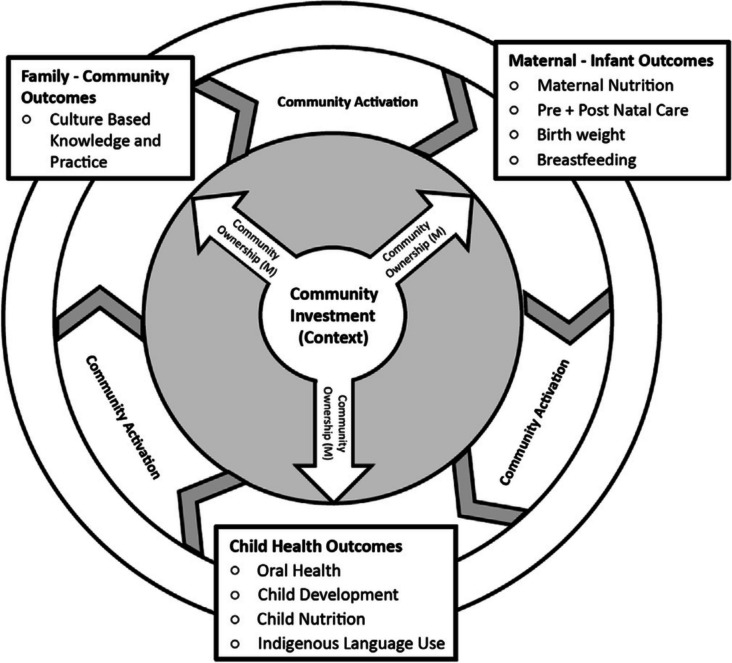
Mid-range theory of Indigenous community investment-ownership-activation

When successfully implemented, OHC projects model a collective and living animacy and energy that builds on and reflects what the lead author has described elsewhere as “community activation” (Smylie et al., 2016). “Community activation” describes a sustained and collective set of actions in which local Indigenous community is dynamically engaged and working together on a task. It was identified as a key cross-cutting outcome in a mid-range theory of “community investment-ownership-activation” that was developed and tested during a realist examination of Indigenous leadership and participation in prenatal and infant toddler health promotion programming (Fig. 1) (Smylie et al., 2016). In keeping with many Indigenous languages, “community activation” is a verb-based concept with roots in the Cree term “e-miyo-mamawi-atoskatamahk” which can be “re-presented” in English as “working together in a good way on a task” (Smylie et al., 2016). In the case of OHC, the task is by community, for community health assessment and response.

The achievement of “e-miyo-mamawi-atoskatamahk” or community activation is understood to be conditional upon achieving a contextual “tipping point” such that the overwhelming perception of local Indigenous community members is that the project is derived from and intrinsic to local Indigenous community (versus externally imposed) and that the act of participating in OHC represents an expression of Indigeneity as it is locally understood. This tipping point mechanism is labelled “community ownership” (Smylie et al., 2016). Within the context of OHC, the type of ownership we speak of is aligned with that of OCAP® in which ownership is understood as “the relationship of First Nations to their cultural knowledge, data, and information” (First Nations Information Governance Centre, 2019).

We understand the context in which “community investment” is achieved as one in which a threshold level of local Indigenous personal and/or collective commitment to and support for (both attitudinal and material) the program (in this case, the local OHC project) has been reached (Smylie et al., 2016). Core OHC methodologic principles and linked application strategies that support the achievement of “community investment” and subsequently trigger “community ownership” and “activation” among Indigenous and allied individuals and organizations in local Indigenous communities are detailed in the next section.

### Core OHC principles

**Fig. 2:**
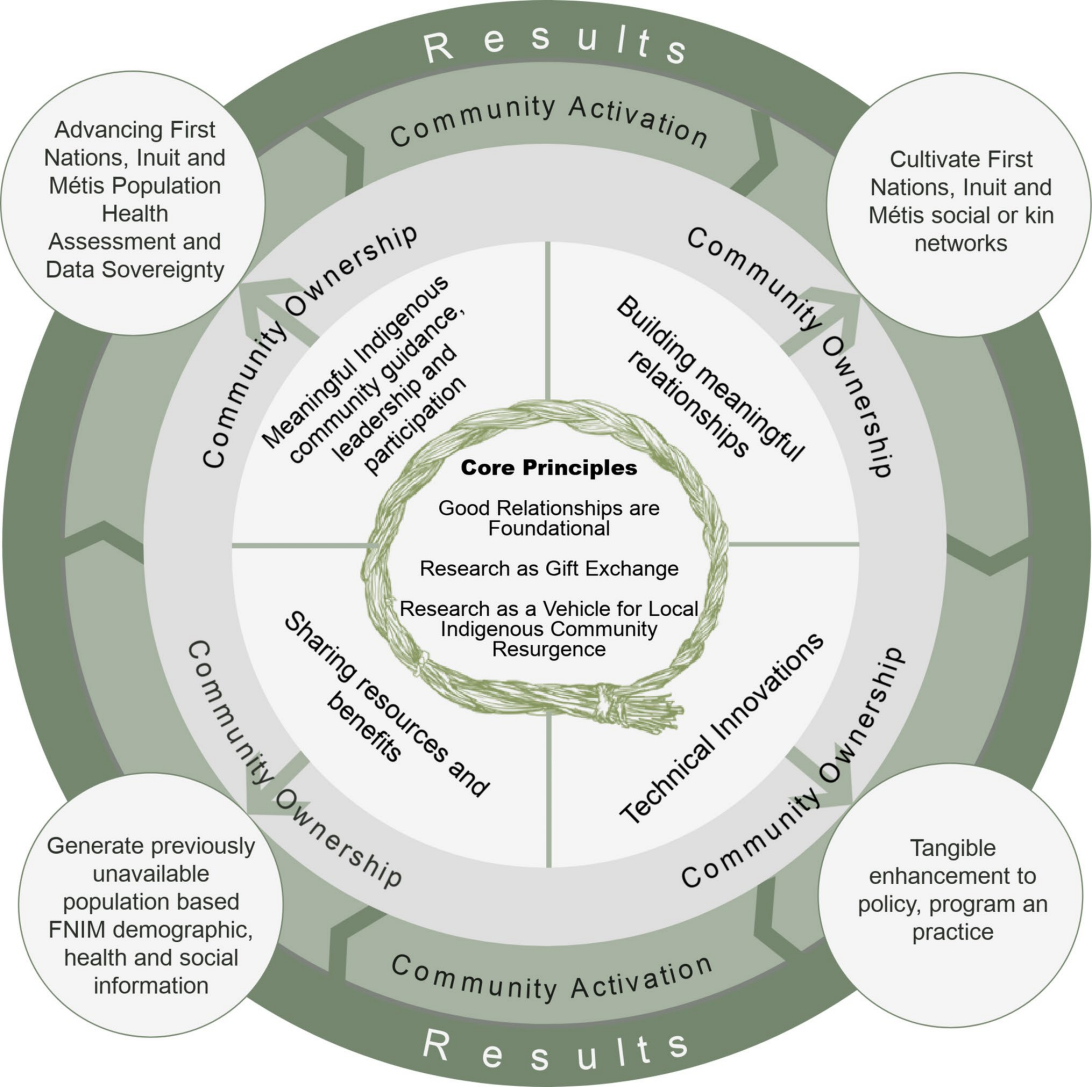
OHC core principles and key implementation strategies

Upon reflection, there are three core principles or applied operational premises that provide the scaffolding for the more specific and concrete OHC implementation strategies. These are the following: *good relationships are foundational*; *research as gift exchange*; and *research as a vehicle for local Indigenous community resurgence* (Fig. 2). Like the three strands in a braid of sweetgrass, each of these principles may be considered a “bundle” of intertwined and interconnected concepts and premises that can be unpacked, reflected upon, and experientially applied.

*Good relationships are foundational* aligns with the Cree principle of “miyo-wîcêhtowin” which can be represented in English as “getting along well with others, good relations, expanding the circle” (Cardinal & Hildebrandt, 2000). The traditional Cree understanding and application of this principle sets a very high bar for individual and collective conduct, for according to this law, humans are required to conduct themselves in a manner that contributes to positive or good relationships not only with people, but with all things, in a manner that is both practical and sacred. Essentially, everything in creation is considered a relative to be treated with kindness, respect, and dignity. While specific teachings regarding relationships and kinship vary across FNIM communities, good relationships are of cross-cutting and foundational importance, which is why this is a core principle of the OHC methodology.

Application of this principle to applied health research requires a reframing of the relationships between what in non-Indigenous research paradigms and contexts are understood as the “academic research”, “community research partner”, and “community research participant” into a large and ever-expanding and open circle of community relatives, each of whom has a role and responsibility in the collective action being undertaken—in this case, the local OHC project. Just like any “extended family”, members of the OHC research team need to be clearly self-located within the local community “kinship structure” and positive pre-existing relationships with “earned trust and respect” based on previous positive relational experiences are key. As a result, strong existing FNIM community engagement skills, lived Indigenous experience, and a commitment to ongoing learning and self-development with respect to FNIM ways of knowing and doing are prioritized in OHC leadership and human resources strategies.

The principle of “miyo-wîcêhtowin” includes the teaching of “expanding the circle” (Cardinal & Hildebrandt, 2000). To operationalize this instruction, OHC projects aspire to support an inclusive and collaborative approach that is respectful of the significant diversity within and across the FNIM nation groups represented in cities.

The framing of *research as gift exchange* is rooted in the principle of reciprocity or sharing in relationships. It is also tied to upholding “miyo-wîcêhtowin”, since positive interpersonal and inter-nation exchange is key to establishing oneself as a good relative and essential to the development and maintenance of “wahkotowin” or kinship (Thistle & Smylie, 2020; Koleszar-Green, 2018). In the words of Elder Maria Campbell: “Family (to our old people) meant sharing all things – wealth, knowledge, happiness, and pain.” (Macdougall, 2017, p. 9). With respect to OHC projects, the principle of reciprocity requires the framing of the research as a co-production of knowledge that involves the “family” of Indigenous and allied scientists, Indigenous health service partners and collaborators, and research participants who can all learn from each other. Each individual from across these groups is understood to hold a valuable piece of the research “puzzle” and it is understood that the collective bringing together of these individual contributions will generate much more than what any one individual or subgroup of individuals could on their own. The process of gifting knowledge(s), experience, skills, resources, and commitment to the OHC project for the benefit of the larger community is aligned with Indigenous teachings regarding sharing (Saskatchewan Indian Cultural Centre, 2009). Implementation of the principle of reciprocity or sharing in relationship facilitates Indigenous community social accountabilities across research processes, since it is understood that there needs to be reciprocal sharing of resources and collective benefit.

The conceptualization of *research as a vehicle for local Indigenous community resurgence* is a core aspect of the community investment-ownership-activation theory of success described above. Local Indigenous community members need to feel that actively participating in OHC projects is aligned with the expression of their Indigeneity and will contribute to improving the well-being of the community. Given the troubled and often violent history of biomedical research in Indigenous community contexts (Smith, 2021; Mosby, 2013), advancement of a premise that research can advance Indigenous sovereignty and interests requires thoughtful messaging by trusted Indigenous community leaders and strong pre-existing and ongoing local community engagement. Key to the realization of this principle is the leadership role taken up by Indigenous health service providers in the implementation of OHC project participant recruitment and subsequent survey interviewing. These Indigenous health service providers have existing demonstrated Indigenous community track records of providing Indigenous-specific models of care that are strongly rooted in local Indigenous ways of knowing and doing, which in turn is based on a core understanding that linking active expression of Indigeneity with health services and programming is key to advancing Indigenous health and well-being.

Another key element that upholds the conceptualization of OHC as a *vehicle for local Indigenous community resurgence* includes assuring a visible, representative, and leading Indigenous presence at all levels and in all aspects of OHC projects, including the research and community implementation teams and all project messaging.

### Key implementation strategies and tools

Several key strategies and tools support OHC project implementation in a manner that is aligned with the methodology described above. Noting that a detailed description of the myriad of ways that the Indigenous paradigm and principles described above are translated into the day-to-day practical implementation of OHC projects is beyond the scope of this paper and best understood experientially, we have grouped these implementation strategies into four categories, which we describe below: (1) active building and maintaining of relationships; (2) meaningful Indigenous community guidance, leadership, and participation in all aspects of the project; (3) transparent and equitable sharing of project resources and benefits; and (4) technical innovations (Fig. 2).

As described above, OHC project success is understood to be linked to positive relational experiences within and across partnered and collaborating organizations and for all study participants. This requires active and ongoing attention to *building and maintaining relationships*. With respect to implementation, it is important to note that for the most part, OHC projects build on pre-existing “within community” relationships in which there is already some sharing of the Indigenous values and principles that underpin the OHC methodology. At the leadership level, the research relationships are formalized at project initiation. This provides an opportunity to clarify and refine these shared operating principles.

A similar process occurs as part of Indigenous community participant recruitment and subsequent survey interviewing, where recruitment and survey interview staff engage study participants as part of the process of recruitment and informed consent. To optimize relational and cultural attunement in recruitment and interview processes, community implementation and interview staff are commonly members of the local FNIM community from which participants are recruited, and lived Indigenous community experience is a pre-requisite for hiring. Survey interviewer training includes practice with each other and FNIM community members who do not meet study inclusion criteria (i.e. live outside the study catchment area). This provides an opportunity for direct experiential feedback to interviewers regarding their interviewing technique. Once relationships are established, structured and culturally relevant relationship maintenance activities across the web of project relationships include structured and unstructured team and leadership “check-ins”; structured team-building activities for community project staff during recruitment and interviewing phases (for example, weekly crafting, debriefing, and self-care sessions); active solicitation of local Indigenous community project feedback from participants and collaborating organizations; meeting and communication systems that support prompt response to community partner, participant, and/or staff concerns or suggestions; and support of project leadership and staff by knowledge keepers, Elders, and ceremony. Finally, careful, frequent, and ongoing communication regarding tasks, timelines, deliverables, and resources at the governance and operational levels is essential to maintaining good relationships, especially given the complexity of OHC projects.

Built-in strategies to align OHC implementation with the notion that our kinship circles need to be open and inclusive entail purposeful mapping of, and outreach across, gender, age, nation, and occupational, housing, and family status in our initial selection of sampling respondent-driven sampling (RDS) seeds^2^ and ongoing efforts to ensure that the project staff and leadership represented the diversity of FNIM involved in the local project.

While OHC Indigenous community partnership models have been iteratively refined and locally customized over progressive projects, there are several common structural components and strategies that support *meaningful Indigenous community guidance, leadership, and participation in all aspects of the project*. The implementation of every successful OHC project has been led or co-led by an established local Indigenous health service agency, who also acts as OHC data custodians. At project initiation, OHC research, data-sharing, and publication agreements are negotiated and signed by the academic research team and the partnering Indigenous health service agency. These agreements facilitate enduring, positive relationships and equitable sharing of resources between the academic and community research partners, and clearly establish core values, Indigenous data governance, specific project roles and responsibilities, and equitable project financing. Recognizing the importance of local community engagement that is inclusive and respectful of the diversity of Indigenous and allied agencies involved in Indigenous health in large urban centres, another key governance strategy is the initiation of a local OHC project reference group or steering committee, comprised of a broader circle of local and related Indigenous and allied health and social service agencies. Successful implementation of this strategy involves regular facilitated meetings of these circles to gather input on and support for project implementation including local tailoring of the survey tool; supported local data governance; and informed project dissemination drawing on local policy and practice priorities. These governance processes ensure that research, data collection, analysis, and dissemination are strongly aligned with existing local Indigenous community and agency priorities, which in turn contributes to ongoing Indigenous community project buy-in and participation.

Another core implementation strategy is that of *transparent and equitable sharing of project resources*, including but not limited to funding and staffing. This is closely tied to the principle of reciprocity in relationships. Equitable community implementation of OHC projects in a context in which urban Indigenous health and social service providers are commonly strikingly and chronically under-resourced and under-staffed requires careful and upfront identification of required infrastructure, material, and staffing costs at the time of proposal development. It also requires expert navigation of research funding mechanisms to ensure adequate funding. Specific strategies include sharing details of project budgets and funding received with Indigenous implementation partners; prioritizing Indigenous community resource and staffing needs; paid secondment of community project executive leads to ensure they have protected time for project leadership; and community incentives for participation. In general, the vast majority of OHC research funding is directly allocated to community implementation partners. An ongoing commitment to reciprocal skills building across the research and community implementation team takes the form of structured and unstructured training, workshops, advanced conversations regarding methods, implementation analysis, and dissemination, and co-production of reports, presentations, and manuscripts.

Key *technical innovations* of OHC include the adaptation of respondent-driven sampling (RDS) methods to recruit representative cohorts of FNIM living in cities; the development and implementation of tailored *respectful community health survey tools* at each OHC site; and the linkage of OHC cohorts to the comprehensive health service use data holdings at ICES (formerly the Institute for Clinical Evaluative Sciences). ICES is an independent, non-profit agency that supports health services and population health research using data collected through the routine administration of Ontario’s system of publicly funded health care. In addition to advancing the scientific value of OHC studies, each of these technical innovations facilitated study implementation in alignment with the underlying OHC Indigenous methodology and principles. For example, the application of RDS, a recruitment method that draws on social networks to understand population characteristics, is strongly aligned with Indigenous relational, kin-based approaches, systems, and accountability in that it levers and applies community social networks to generate and interpret data.

As we were working on the survey tool for the first OHC study, a community research co-lead aptly reconceptualized what our research team was calling the “rapid health assessment survey” as the “respectful health assessment survey”. We had been building on non-Indigenous evidence suggesting that survey tools need to be kept short and conducted quickly to optimize participation and minimize response burden on participants—hence the concept of “rapid” (Graf, 2008). By applying a local Indigenous paradigm to the survey interaction, this community research co-lead helped us understand that if we approach participants in a relational manner and conceptualize the interview as an opportunity for “keeoukaywin” or visiting (Thistle & Smylie, 2020), then the process need not be rushed and a longer and more detailed conversation can take place.

Linkage of OHC cohorts to ICES health service use data holdings is important in that it addresses the absence of FNIM identifiers in these data holdings; ensures that local FNIM individuals and communities control and determine the manner in which FNIM identity is determined; generates previously unavailable population-based FNIM health service information; and creates an opportunity to follow FNIM community health and health service access over time in a context of high FNIM mobility. By advancing “by community, for community” FNIM identity in these datasets and generating FNIM community health information that is owned and controlled by FNIM leaders and can be used to advance FNIM collective health interests, the ICES linkages align with the conceptualization of research as a tool to advance community resurgence. Specific technical details regarding the implementation of these methods are beyond the scope of this methodology focused paper and are detailed elsewhere (Firestone et al., 2014; Smylie et al., 2018, 2022).

## Results

The OHC methodology has been successfully implemented in six Ontario cities (OHC First Nations Hamilton, OHC Inuit Ottawa, OHC Toronto, OHC London, OHC Thunder Bay, and OHC Kenora) and a seventh OHC project is in progress in Manitoba (OHC Winnipeg). Academic and community research co-leads have worked together with the broader circle of collaborators to actively translate OHC project findings into local practice and service responses; local, provincial, national, and international presentations to community, academic, policy, and practice audiences; and community reports, fact sheets, and academic publications. The significant and growing outputs of the OHC research program are comprised of large and comprehensive sets of previously unavailable, Indigenous community–governed, descriptive, and analytic population health information detailing the demographics, health outcomes, and health service use of FNIM living in urban and related homelands. In keeping with the methodological focus of this manuscript, our focus here is on reporting system-level OHC impact for FNIM living in cities. These system-level impacts include *the advancement of population health assessment and data sovereignty infrastructure and capacities for FNIM living in cities and beyond*; *recognition and cultivation of FNIM social or kin networks at the individual and organizational levels*; *addressing demographic, health, and social data and information gaps for FNIM living in cities*; and *advancing tangible enhancements to programs and services*. Specific FNIM health information findings are published elsewhere (Firestone et al., 2014; Smylie et al., 2011, 2018, 2022; Smylie & Firestone, 2017; Rotondi et al., 2017; Kitching et al., 2020; Well Living House, 2023a, 2023b; Southwest Ontario Aboriginal Health Access Centre, 2023; McConkey et al., 2022; Snyder et al., 2022a, 2022b, 2022c).

### Advancement of population health assessment and data sovereignty infrastructure and capacities for FNIM living in cities and beyond

The six existing OHC datasets represent the first population-level, Indigenous community owned and controlled, longitudinal health and social databases that inclusively and comprehensively describe demographics and health determinants, outcomes, and service access for First Nations, Inuit, and Metis living in urban and related homelands. The six cohorts collectively include 4393 FNIM individuals, representing a combined total population of > 170,000 FNIM living in urban and related homelands, at the time of baseline survey (Table 2).

**Table 2:** Our Health Counts sample size summary

Our Health Counts study site	Year	Total sample size	Adult sample size	Child sample size	Conservative^a^ population estimate [95% CI] (year)	Non-conservative population estimate [95% CI] (year)
Hamilton First Nations	2010	777	555	222	N/A	N/A
Ottawa Inuit	2010	504	345	159	1505* [1077–2270] (2011)	3361* [2309–4959] (2011)
Toronto	2016	1150	916	234	62,737 [50,890–82,081] (2016)	84,187 [65,315–118,761] (2016)
London	2016	754	508	246	22,673 [17,822–31,154] (2016)	29,361 [22,060–44,360] (2016)
Thunder Bay	2019	830	601	229	19,652 [16,491–24,268] (2016)	42,269 [31,858–62,777] (2016)
Kenora	2021	378	320	58	8448 [5582–17,377] (2016)	12,892 [7515–45,342] (2016)
Total		4393	3245	1148		

The population surveyed for Hamilton were individuals who self-identified as First Nations. The population surveyed for Ottawa were individuals who self-identified as Inuk. The populations surveyed for Toronto, London, Thunder Bay, and Kenora were individuals who self-identified as First Nations, Inuit, and/or Métis

^a^Don’t know/don’t remember/unreliable assumed to have completed the census. Our working assumption is that most FNIM adults do remember if they did the census, so at the instructions of our community partners, we use the non-conservative population estimates

^*^Population estimates for Inuit in Ottawa are for adults only (15+). All other estimates are for adults and children

OHC research, data, and publication agreements have drawn on existing platforms of Indigenous health and social data governance and management (First Nations Information Governance Centre, 2019; Inuit Tapiriit Kanatami, 2018; Inuit Tapiriit Kanatami & Nunavut Research Institute, 2006; National Aboriginal Health Organization, 2011). In partnership with urban FNIM service provider, we have worked to advance and customize Indigenous data governance tools and processes to ensure that FNIM living in cities, and the Indigenous health and social service organizations that serve them, are represented and included in regional, national, and international efforts to implement Indigenous data sovereignty.

The OHC First Nations Hamilton data-sharing agreement between ICES, the project’s Governing Council, and St. Michael’s Hospital was signed on January 20, 2010 (Smylie et al., 2011, p.94), and represents the first data-sharing agreement between ICES and Indigenous organizations. This agreement structures Indigenous governance of the Indigenous data shared with and generated by ICES in this project. ICES subsequently has negotiated multiple partnerships with First Nations, Inuit, and Metis organizations that include data-sharing and data governance agreements (ICES, 2023).

With respect to Indigenous health human resources, OHC projects have provided an opportunity for in-depth experiential Indigenous health information skills advancement for > 50 Indigenous community health and social service workers; > 20 graduate students; 5 post-doctoral fellows; > 15 Indigenous and allied academic research staff; and 5 new investigators. Additionally, each of the 4393 OHC participants experienced “by community, for community” comprehensive health assessment and response. The success of recruitment, the high rates of survey question completion, and the high rates of participant agreement to ICES data linkage provide evidence that participants felt safe and secure with our community implementation processes.

Finally, OHC projects have contributed to the advancement of RDS methods more generally, including the enhancement and/or validation of RDS estimator techniques (Avery et al., 2019, 2021) and multivariable regression methods (Avery et al., 2022).

OHC projects have *advanced recognition of and actively cultivated FNIM social or kin networks at the individual and organizational levels in urban contexts.* The six OHC RDS recruitment chains represent some of the world’s largest RDS-recruited samples, and RDS recruitment processes overall were brisk and robust. Since the success of RDS recruitment is hinged on the pre-existence of social networks, the repeated success of RDS methods across the six OHC sites provides compelling evidence of strong and resilient FNIM social or kin (as they are understood from an Indigenous paradigm) networks in these cities.

Additionally, OHC projects purposefully cultivated local Indigenous social or kin networks at each site at the individual and organizational levels. Inherent in OHC sampling methods and organizational level partnerships and networking was the recognition that to strengthen local relational networks would be a legacy contribution to local FNIM communities. We recognized from the outset that the strongest way to engage participants in peer recruitment would be through a positive report about the study experience from the person recruiting them and that this required positive and culturally attuned participant engagement and interview experiences. We also understood that positive social engagement within an Indigenous social context is itself a “good medicine”. While the full scope of population-level impacts of OHC on the size and strength of local Indigenous social networks at the individual and organization levels is challenging to quantify, there are many empirical reports of FNIM individuals building new connections with Indigenous service agencies after participation in OHC. Additionally, we had near-complete retention of OHC community-based survey staff to project completion across all project sites. At the organizational level, there are also multiple examples of subsequent academic–community projects and partnerships. For example, in the early months of the COVID-19 pandemic, OHC academic (Well Living House) and community (Seventh Generation Midwives Toronto) partners in Toronto joined a second Indigenous community health service provider (Na-Me-Res) to create a comprehensive COVID-19 assessment and response system for FNIM living in Toronto. The resultant Auduzhe Mino Nesewinong program includes clinical services such as testing, contact tracing, case management, vaccination, primary care, and hospital referrals (We Count COVID-19 Information and Resource Sharing Hub, 2023a) and a COVID-19–focused FNIM community cohort (We Count COVID-19 Information and Resource Sharing Hub, 2023b). Additionally, the Well Living House worked with Indigenous community partners to pivot OHC-ICES data linkage work and produce otherwise unavailable COVID-19 outcomes information for FNIM living in Toronto, London, Thunder Bay, and Kenora (Smylie et al., 2022).

### Addressing demographic, health, and social data and information gaps for FNIM living in cities

When we initiated the OHC research program in 2008, the Canadian Census and Statistics Canada health surveys that used the Census as their sampling frame such as the Canadian Community Health Survey (CCHS) and the Aboriginal Peoples Survey (APS) were the primary source of inclusive population-level health and social information for First Nations, Inuit, and Metis living in urban and related homelands of Canada. FNIM service organizations were regularly reporting that Canadian Census–derived population estimates for these populations were erroneously low. The CCHS and APS were not designed to provide the comprehensive, locally disaggregated, and Indigenous-specific health and social information that is required to optimize the planning, delivery, and evaluation of health services and were underpowered to do so. Linkages of federal registries of “Status Indians” and Metis Registries to provincial and territorial health service use data were in development, as was First Nations and Metis governance and management of these processes and technical refinements. In sum, Indigenous and allied health and social service organizations serving FNIM living in cities were commonly operating without the type of local- and regional-level population health assessment information that is considered essential.

As a result of the OHC projects, comprehensive demographic, health, and social information is now available for FNIM in six cities in Ontario, a province in which over 70% of the FNIM population lives in cities (Statistics Canada, 2022b). Further, OHC research has shown at five sites that the Canadian Census underestimates FNIM population size in cites by a factor of 2–4 (see Snyder et al., this issue). Additionally, OHC study findings suggest that the long-form census and census-linked sociodemographic surveys dramatically underestimate the sociodemographic disadvantage experienced by FNIM living in cities due to a systemic participation bias for FNIM participants living in Ontario cities (see Snyder et al., this issue; Smylie & Firestone, 2015). Finally, as mentioned above, with the onset of COVID-19, we were able to pivot the OHC-ICES data linkage work to produce otherwise unavailable COVID-19 outcomes information for FNIM living in Toronto, London, Thunder Bay, and Kenora (Smylie et al., 2022).

OHC findings have contributed to* tangible program and service enhancements,* including new and/or enhanced funding for Indigenous health and social services for FNIM living in participant OHC cities. For example, OHC First Nations Hamilton findings regarding high rates of homelessness resulted in a decision by the City of Hamilton to double Indigenous-specific funding for homelessness. The former CEO of Dedwadadehsney>s Aboriginal Health Centre, the OHC community partner organization in Hamilton, further credits the OHC Hamilton report as facilitating a tripling of the Centre’s budget and services (personal communication with corresponding author, 2024). Similarly, OHC Toronto findings regarding population size and unmet health and social needs of FNIM children were translated into the release of $12 million of new Indigenous child development funding support by the City of Toronto.

Further, OHC findings have been directly applied to enhance clinical services across all sites. For example, the findings of the OHC Inuit Ottawa study were applied to the development of the new Akausivik Inuit Family Health Team (AIFHT), which has been providing medical care to Inuit in Ottawa since 2014. During the COVID-19 pandemic, the information generated by OHC Toronto, London, Thunder Bay, and Kenora regarding rates of SARS-COV-2 testing, infection, vaccination, and linked hospitalization facilitated tailored, local responses and highlighted the need for ongoing FNIM-focused vaccination efforts and improved access of FNIM to regular primary care providers.

## Discussion

This paper has detailed and explicitly articulated the underlying Indigenous methodology that facilitated OHC successes, using an Indigenous paradigm and conceptual framework. We applied a mid-range theory of how local Indigenous community members can be engaged at the individual and organizational level to create a context in which the community reaches a state of “activation” or “e-miyo-mamawi-atoskatamahk”, in which they are working together in a good way on the project (Smylie et al., 2016). Drawing on traditional Indigenous knowledge and our collective OHC experience, we identified and described three underlying Indigenous principles (*good relationships are foundational*; *research as gift exchange*; *research as a vehicle for local Indigenous community resurgence*) and four linked implementation strategies (*actively building and maintaining relationships*; *meaningful Indigenous community guidance, leadership, and participation in all aspects of the project*; *transparent and equitable sharing of project resources and benefits*; and *technical innovations*) that together provide the pre-requisite infrastructure and context for sustained local Indigenous community engagement and activation in OHC projects. Our results illuminate the key system-level impacts of OHC for FNIM living in cities, including advancement of population health assessment and data sovereignty infrastructure and capacities for FNIM living in cities and beyond; recognition and cultivation of FNIM social or kin networks at the individual and organizational levels; addressing of demographic, health, and social data and information gaps for FNIM living in cities; and tangible enhancements to programs and services.

The purpose of this paper was to detail, from an Indigenous paradigm and viewpoint, how, why, and to what end Our Health Counts projects achieve their overarching goal of “by community, for community” advancement of local First Nations, Inuit, and Metis population health information. In the context of ongoing dialogues regarding Indigenous self-determination in research (Roach & McMillan, 2022) and Indigenous data sovereignty (Lovett et al., 2019; Carroll et al., 2020), which commonly assert principles but less often offer applied and locally detailed applications, we anticipate this will be a useful “how to” guide that is grounded in practice and the local realities of First Nations, Inuit, and Metis living in urban and related homelands in Ontario.

Detailed, Indigenous community‒rooted explications of why, how, and under what circumstances an Indigenous health project or program functions, which are framed by and actively assert Indigenous worldviews and practices, are rare in the published health science literature. This is particularly apparent in quantitative domains, where a positivist frame is commonly assumed. The resultant reduction in the social value of health and public health scholarship for Indigenous communities manifests in multiple ways. Gaps in the explicit articulation and reconciliation of positivist paradigm assumptions with the complex realities of Indigenous communities located in settler states commonly result in a deficit-based framing of Indigenous health and well-being that implicitly draws on and reinforces biased colonial beliefs of Indigenous inferiority and masks root challenges, such as coloniality and the ongoing unequal distribution of health and social resources (Watego et al., 2021). For example, epidemiologic models exploring population health inequities commonly include Indigenous identity as a “risk factor”, even though evidence demonstrates that the root causes of these inequities include anti-Indigenous racism and disproportionate Indigenous social deprivation (Harris et al., 2006).

From a methods and implementation science perspective, failure to recognize and detail the specifics of local Indigenous community context, engagement, and participation as a core component of study documentation not only side-lines Indigenous community contributions, but also restricts effective reproducibility, as according to our mid-range theory, these elements are the critical ingredients for success. The challenge is that these essential local Indigenous principles and implementation strategies are commonly implicitly understood by local Indigenous community project staff but may be hidden to outsiders or newcomers to a local Indigenous community, especially if they are primarily operating in a non-Indigenous paradigm. Yet, it is often outsiders or newcomers who lead academic project research and reporting, since these systems are not accessible to local Indigenous community project staff. As a result, the underlying local Indigenous ingredients for success may be stripped away, unrecognized, or misrepresented in study reporting. Subsequent attempts to reproduce or “scale up” the project or program will only succeed if new local sites have Indigenous project staff who are able to build in local Indigenous principles and processes.

The difficulty of validly representing Indigenous principles and practices in written English (or other non-Indigenous languages) and existing academic health sciences research, editorial, and publishing systems, which are dominated by non-Indigenous people, principles, and practices, compounds this problem. These establishments regularly essentialize or underestimate the complexity and diversity of Indigenous knowledge(s) and practice and institutionalize “short-cut” approaches to and tools for bridging Indigenous/non-Indigenous epistemic gaps. For example, the trust relationships required for “miyo-wicehtowin” or good relations are commonly earned over time through repeated demonstrations of reciprocal caring and sharing that are of tangible collective benefit and can take many years to establish. A short course in Indigenous cultural safety or Indigenous research ethics alone is most likely insufficient for those who are outsiders or newcomers to Indigenous communities to successfully implement health research or programs that adhere to the principle of “miyo-wicehtowin”.

While this manuscript is the product of a purposeful attempt to interrupt the processes described above and meaningfully represent the underlying Indigenous principles and practices essential to successful OHC projects, because it is in the form of a written English word–limited academic publication, it too is inadequate. The cultural integrity of the representation is further hindered by the fact that while it is written by a group of Indigenous and allied academics and community service providers with diverse social locations and worldviews, we do not fully represent the multiplicity of experiences and perspectives of all the FNIM individuals and communities that participated in OHC. Finally, as noted earlier, the articulation of the underlying Indigenous theory and principles relies heavily on the specific Metis-Cree worldviews of the two first authors. Our intention was to avoid an essentialized pan-Indigenous discussion and stimulate additional nation-specific discussion and dialogue that assert diverse FNIM and international Indigenous paradigms and languages.

To quote former Assembly of First Nations national chief Phil Fontaine: “the answers lie in our communities” (Smylie & Phillips-Beck, 2019, p. E207). This holds true not only with respect to understanding OHC methodology, but also for the majority of OHC health and social information findings. The methodology is best understood through local community immersion before, during, and after project implementation. Likewise, much of the “newly” available information about FNIM living in urban and related homelands that was generated by OHC projects was already “known” by local Indigenous community members. For example, multiple Indigenous community co-leads told us at the initiation of the OHC research program that the Canadian Census was significantly underestimating the size of the Indigenous communities in local cities and this was a key rationale for project development. Additionally, many of the other demographic, social, and health characteristics of local FNIM populations in OHC cities were accurately described by local Indigenous members prior to the study. The gap that OHC projects filled was really with respect to the type of evidence that would be perceived as relevant to non-Indigenous policy makers, who required a “re”-presentation of what local Indigenous community leaders already knew in a format that they considered valid—a statistically representative population-level database. Our work here is therefore really an inadequate “re”-presentation of mostly pre-existing community knowledge and processes to the larger audience of health researchers, policy makers, and practitioners.

Our examination of the system-level impacts of OHC projects highlights the tangible Indigenous community social value of both project outputs and processes. Key outputs include the generation of previously unavailable population-based FNIM health information, which in turn directly contributed to enhancements of policy, funding, and service delivery. Process outputs included strengthening of community kin networks and the local advancement of Indigenous data sovereignty. Specifically, as local communities were supported to develop and apply their own health information systems (HIS), negative perceptions of HIS linked to previously harmful colonial processes were transformed and health information became increasingly understood as a local community asset. By the end of each initial project cycle, community leaders and research partners were speaking confidently about project data outputs. From an Indigenous community advancement perspective, these process outcomes may represent the more important and lasting project contributions. They are also directly linked to and facilitated by our methodological approach which explicitly and purposefully elevates and builds on local Indigenous community priorities, needs, and strengths.

The broader colonial context in which the OHC projects are situated provides additional limitations and challenges for the OHC methodology. As noted in the Introduction, FNIM living in urban and related homelands in Canada are commonly discounted in enumeration, needs assessment, policies, programs, and service agreements. Bridging these gaps in basic social recognition and infrastructure through research-funded initiatives is clearly insufficient. Longer-term investment and systems transformation, such that FNIM are counted into HIS, policy, services, and programming across geographies, is required. Unfortunately, this exclusion of FNIM living in cities is still very much the status quo for many policy makers, practitioners, and researchers and is embedded into government, health, and public health systems at the local city, provincial/territorial, and federal levels. This challenging landscape has actively interfered with OHC project implementation and results dissemination, including publication and policy uptake of project results. It is our hope that our health policy, practitioner, and research peers will continue to join us in problematizing the discriminatory exclusion of FNIM living in cities and recognize the innovation, quality, and strength of our methodologies.

Additionally, colonial contexts, by definition, are rooted in non-Indigenous values, principles, and processes. As a result, the implementation of Indigenous research processes supported by partnerships between non-Indigenous funding and academic institutions and Indigenous service agencies requires ongoing vigilance and harmonization. Collisions between knowledge systems and implementation approaches should be anticipated and 

## Conclusion

Our Health Counts was envisioned and designed to elevate and support First Nations, Inuit, and Metis community well-being in urban and related homelands. The OHC methodology, developed in partnership with local FNIM health service organizations and leaders, is rooted in Indigenous principles, strategies, and local systems. This approach has been demonstrated as effective, culturally relevant, impactful, and scalable across diverse Ontario cities with respect to both generation of population-representative health information and advancement of local FNIM community priorities and interests in the domain of health and beyond.

## Contributions to knowledge

What does this study add to existing knowledge?

- Depth description of a ***quantitative*** Indigenous methodology, including Indigenous theory, key principles, implementation strategies, and system-level impacts.
- Application, assertion, and detailing of rooted Indigenous worldview, knowledge, concepts, and practices in a ***quantitative*** academic manuscript.
- Demonstration that Indigenous-governed, Indigenous-led, community-partnered, and community-implemented population health assessment and response in urban and related homelands can be effective, impactful, and of tangible Indigenous community benefit.

What are the key implications for public health interventions, practice, or policy?

- The discriminatory exclusion/marginalization of constitutionally recognized FNIM peoples from quality population-based health assessment and response based on their residency in urban and related homelands is unacceptable and needs to be actively addressed by public health policy makers, practitioners, and service providers.
- Local context is important and needs to be considered in FNIM public health, practice, and policy.
- Shifting from consultation and participation towards FNIM governance, leadership, and management in the development and implementation of health and public health information and response systems is both aligned with current policy and international law and also effective in generating high-quality information and translation of this information into better policy, practice, and health outcomes.

## Footnotes

1 ^1^The word Canada comes from the Iroquoian word “kanata”, which means village or settlement (Olson & Shadle, 1991, p.109).

2 Respondent-driven sampling (RDS) is a sampling method that draws on social networks and their characteristics for recruitment and the generation of population-based estimates (Heckathorn, 1997). We have detailed the technical aspects of its application to Our Health Counts projects elsewhere (Firestone et al., 2014; Rotondi et al., 2017; Smylie et al., 2018; Avery, 2021).
